# Feature Extraction from Building Submetering Networks Using Deep Learning

**DOI:** 10.3390/s20133665

**Published:** 2020-06-30

**Authors:** Antonio Morán, Serafín Alonso, Daniel Pérez, Miguel A. Prada, Juan José Fuertes, Manuel Domínguez

**Affiliations:** Grupo de Investigación en Supervisión, Control y Automatización de Procesos Industriales (SUPPRESS), Esc. de Ing. Industrial, Informática y Aeroespacial, Universidad de León, Campus de Vegazana s/n, 24007 León, Spain; saloc@unileon.es (S.A.); dperl@unileon.es (D.P.); ma.prada@unileon.es (M.A.P.); jj.fuertes@unileon.es (J.J.F.); manuel.dominguez@unileon.es (M.D.)

**Keywords:** deep learning, convolutional neural networks, autoencoders, submetering networks, power consumption

## Abstract

The understanding of the nature and structure of energy use in large buildings is vital for defining novel energy and climate change strategies. The advances on metering technology and low-cost devices make it possible to form a submetering network, which measures the main supply and other intermediate points providing information of the behavior of different areas. However, an analysis by means of classical techniques can lead to wrong conclusions if the load is not balanced. This paper proposes the use of a deep convolutional autoencoder to reconstruct the whole consumption measured by the submeters using the learnt features in order to analyze the behavior of different building areas. The display of weights and information of the latent space provided by the autoencoder allows us to obtain precise details of the influence of each area in the whole building consumption and its dependence on external factors such as temperature. A submetering network is deployed in the León University Hospital building in order to test the proposed methodology. The results show different correlations between environmental variables and building areas and indicate that areas can be grouped depending on their function in the building performance. Furthermore, this approach is able to provide discernible results in the presence of large differences with respect to the consumption ranges of the different areas, unlike conventional approaches where the influence of smaller areas is usually hidden.

## 1. Introduction

In developed countries, power consumption from buildings has steadily increased in the last few decades and this growing trend will continue in the future. It is due to, among others, the improvement of building services and comfort levels and the increment in time spent inside buildings. As a consequence, residential and commercial buildings represent up to 32% of the global power consumption [1]. Nowadays, the building sector is one of the main contributors to global power consumption and polluting emissions, even above the transport and industry sectors [2]. Therefore, a good understanding of the nature and structure of energy use in buildings is vital for defining new energy and climate change strategies [3].

In this paper, we propose the development of a submetering network in buildings with the aim of understanding the consumption patterns corresponding, not only to the main supply but also to each area of the building. The meters comprising the submetering network are in charge of measuring and collecting data used to analyze the consumption profiles. We also propose a methodology for extracting features from power consumption profiles of submeters. That methodology combines convolutional neural networks and an autoencoder in order to extract the relevant features that determine the consumption profile at a higher point in the submetering hierarchy, specifically at the main supply point, where energy billing is carried out.

The main contributions of this paper are:

- The development of a submetering network for a hospital building containing 37 electrical meters communicating with Modbus protocol.
- The proposal of an approach based on deep learning to extract relevant features from power consumption profiles of submeters and to detect their influence on the main supply point, since the aggregation of the relevant features in submeters can be useful to build the global supply profile.
- The validation of the proposed approach using the submetering network at the León University Hospital.

This paper is structured as follows: Section 2 provides the background and literature review. Section 3 presents the developed submetering network at the León University Hospital building. In Section 4, the deep learning approach based on convolutions and an autoencoder is explained. Results of data analysis are presented and discussed in Section 5. Finally, the conclusions are exposed in Section 6.

## 2. Background

The use of energy in buildings should be analyzed in detail, previously to define appropriate management and maintenance strategies. Besides analyzing the whole energy consumption billed by the energy supplier, partial consumptions in certain areas of the building should also be considered. Energy bills can be used as an information source in order to disaggregate the power consumption of different areas and analyze the energy use in buildings [4]. However, the installation of a smart energy meter can provide insightful information about power consumption profiles, not only how much energy is consumed. Besides measuring power consumption, a smart energy meter can exchange information on electricity, water or gas use or even with other energy meters [5].

Non-Intrusive Load Monitoring (NILM) techniques [6] are able to disaggregate power consumption and discern devices or demands from the aggregated data acquired from the main energy meter [7]. NILM techniques include traditionally six phases: data acquisition, data processing, event detection, feature extraction, event classification, and energy computation [8]. NILM enables the analysis of the total power consumption through signal processing algorithms, which are applied to the collected data from a single point in order to achieve an improved demand-side energy usage [9]. After applying NILM techniques, some partial components, which add up to the original measured signal, are obtained. The combination of decomposition and visualization methods could let users discover relevant temporal patterns, which otherwise may be difficult to interpret [10].

Nowadays, the advances on metering technology (standard and wireless connectivity, reliable measurements, capability of storing data, low-power consumption, etc.) and low-cost devices [11] make the installation of many smart meters in a building feasible to measure not only the main supply point, but also other intermediate points in the energy distribution network or even certain systems using meters only occasionally connected [12]. Therefore, partial consumption measurements within buildings can be determined through energy submeters installed at points of interest [13]. These submeters can be linked together to the energy management system (EMS) or to the cloud, through standard protocols such as Modbus TCP, M-Bus, Zigbee, etc. [14], obtaining a submetering network. These networks benefit from Internet of Things (IoT) technologies to measure, collect, and analyze consumption data, enabling even the integration of buildings into a smart network [15]. Submetering infrastructures are essential to understand the use of energy within buildings, perform daily, and hourly predictions at the main and lower points and project savings due to changes in consumption habits and/or installation of more efficient equipment [13,16]. These infrastructures include the measurement of all energy inputs to the building (electricity, gas, water, etc.) [17] and enable the aggregation of the whole consumption profiles from the submeters. Submetering networks can be also useful for monitoring energy quality, not only for analyzing the power consumption [18].

Submetering networks provide a huge amount of data which can be useful to tackle energy efficiency in buildings [19]. Analyzing data can be also useful for detecting faults in equipment, finding opportunities for load shifting, revealing poor building systems and processes, identifying inappropriate building automation strategies, etc. [20]. Although these data contain valuable information, it is difficult to extract it with conventional techniques [21]. Considering the number of submeters, the hierarchy comprising these architectures and the set of variables they provide, typically with nonlinear relationships among them, dimensionality reduction techniques [22] are required to project the whole variables on a low-dimensional latent space. The energy use in a building could be summarized by the latent variables in this low-dimensional space. Dimensionality reduction algorithms such as t-SNE [23] have been applied to address this purpose. In [24], it is used to project daily electricity consumption profiles of several building and detect correlations with the environmental conditions.

Furthermore, data from the submetering networks could contain redundant and irrelevant information, given their hierarchical structure. Thus, a feature extraction process should be carried out in order to obtain meaningful features, with an influence on the consumption at the main supply point, i.e., the billing point that determines the energy use in the whole building. Novel deep learning algorithms [25,26] can be applied for feature extraction in submetering networks. Indeed, these algorithms have been already applied satisfactorily in other research fields such as image processing [27], since they have a huge potential for that task. In this sense, convolutional neural networks (CNN) [28] are based on the repeated application of the same filter to the input data resulting in a feature map (comprising the activations). They have the ability to learn several filters in parallel, enabling the extraction of abstract and non-trivial features in the input data. Convolutions can be separated across their spatial axes by dividing a kernel into two smaller kernels, so that computational complexity decreases, favoring generalization and facilitating the convergence. A separable convolution comprises two consecutive stages: the depthwise and pointwise convolutions [29]. The first (depthwise) convolution is performed over the spatial dimensions (width and height of the data), whereas the second (pointwise) convolution is performed over the number of channels of data [30]. These two sequential convolutions produce the same result as a standard convolution, but they provide new intermediate feature maps for each channel and the network runs faster.

Additionally, autoencoders (AE) [31], which attempt to reproduce its input in its output, while passing through a reduced encoding, can be used for feature extraction. For that purpose, we can use the compressed representation of the input data produced by the encoder function at the bottleneck, before a decoder produces the reconstruction of the input data from the encoded representation, so that it is as close as possible to the original input. Standard (AE) and stacked (SAE) autoencoders have been used effectively for feature extraction in images [32]. Representative reduced features can be obtained in deep layers using SAE. However, autoencoders (AE) could reveal some minor or insignificant features when input data are too heterogeneous. On the contrary, denoising autoencoders (DAE) [33] are more robust than standard autoencoders since they learn to remove noise in the input data, besides compressing data. For that purpose, a denoising autoencoder corrupts the input data by randomly setting some of the input values to zero. This method has been already applied to denoising problems in image processing [34]. Moreover, denoising autoencoders (DAE) could be used in order to tackle a Non-Intrusive Load Monitoring (NILM) approach. In this case, the power consumption of the main supply in an installation would be the noisy signal and the power consumption of the intermediate points to be disaggregated would be the filtered signal. Thus, a DAE could identify the electric load in the intermediate points of the distribution network.

Deep learning networks combining the above-mentioned methods can be also useful to analyze data from submetering networks. In this sense, a Convolutional Autoencoder (CAE) is an autoencoder in which the nonlinear transformation is obtained by a convolutional neural network. Using CAE, data in the time domain can be converted into a representative vector in a lower dimensional encoded space, preserving the features in power consumption patterns [35]. Convolutional Variational Autoencoder (CVAE) is also proposed to learn automatically features in raw data and achieve energy disaggregation [36]. It combines convolutional neural networks and a variational autoencoder (VAE), which comprises a probabilistic encoder and a generative decoder. The encoder makes use of convolutional layers to learn features of a probability distribution modelling from the aggregated signal and the decoder uses deconvolutional layers to estimate the specific disaggregated signals.

## 3. Submetering Infrastructure

**Figure 1:**
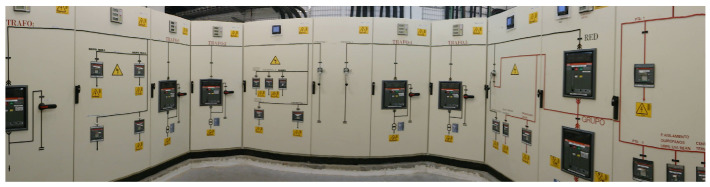
Photo of the output of TC1.

Electricity supply to the León University Hospital is carried out through two 45 KV power lines. One of them is the main supply line and the other is in reserve and it is only used in case of failure or maintenance of the main line. Both lines feed a Main Transformation Centre (MTC), where the voltage is reduced to 13.2 KV. From this point, electricity is distributed to three transformation centers (TC1, TC2, TC3), which reduce again the voltage to 400 V (the nominal value of supply in Spain). A photo of the modules (cells) in TC1 can be observed in Figure 1. The three transformation centers(TC1, TC2, TC3) are linked in a ring with the aim of guaranteeing the electricity supply in the building. Downstream of each of the three transformation centers, there are several distribution electrical panels corresponding to different areas of the hospital.

**Figure 2:**
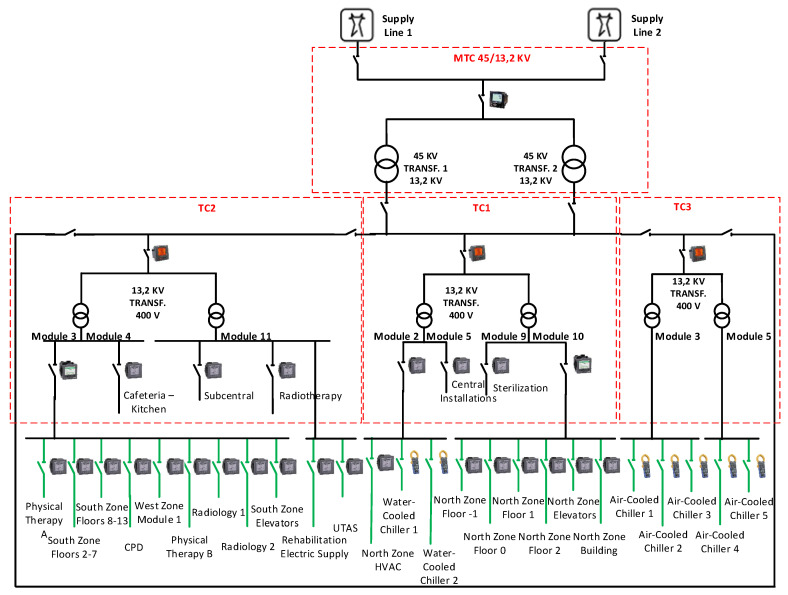
Single-line diagram of the electricity supply and meters at the León University Hospital.

In order to understand the consumption patterns, a submetering network has been designed and developed, installing 30 submeters in the distribution panels. Furthermore, seven other electrical meters have been installed upstream, e.g., in transformation centers TC1, TC2 and TC3 and their output modules (cells), including one at the main supply point (MTC). Figure 2 depicts the single-line diagram of the electricity supply at the León University Hospital with the corresponding electrical meters.

Electrical meters and analyzers measure and store more than 30 variables (voltages, currents, powers, energies, harmonics, etc.) for each of the three phases and neutral of the electricity supply. Some new electrical meters, models ION7650, PM8000, PM5560, and PM5110 by Schneider Electric (https://www.se.com/ww/en/product-category/4100-power-monitoring-and-control/), have been installed all over the building. Moreover, existing meters, model CVM-NRG96 by Circutor (http://circutor.es/en/products/measurement-and-control/fixed-power-analyzers/power-analyzers), and internal meters in Petra and Trane chillers, are also used. Table 1 lists all the meters comprising the submetering architecture in the hospital.

Specifically, an ION7650 meter has been installed in the MTC which measures the whole electricity consumption of the building. This meter is installed at the same point as the billing meter of the electricity supplier, so it is possible to compare both measures. Each of the three TCs is composed of several modules (cells) in which measuring equipment has been installed. In the TC1, an existing meter is used to measure the whole consumption in this center. Furthermore, 4 meters have been installed in Module 2, Module 10, Central Installations, and Sterilization. In the TC2, besides the existing TC2 main meter, there are four other meters installed: Module 3, Cafeteria-Kitchen, Subcentral, and Radiotherapy. The TC3 has an existing meter for the measurement of the total consumption of all the air-cooled chillers. The meters installed in TCs are PM8000 and PM5560 models by Schneider Electric and CVM-NRG96 model by Circutor (see Figure 2).

The distribution panels are dependent on the TC1 feed different areas of the north zone of the building (*Virgen Blanca*) and the water-cooled chillers. PM5560 meters have been installed in the North Zone Building and North Zone Floor-1. PM5110 meters are installed in the remaining areas which are supplied from Module 10 in TC1 (North Zone Elevators and North Zone Floors 0–2). A PM5110 meter has also been installed in North Zone HVAC which is fed from Module 2 in TC1, together with the Trane water-cooled chillers. They incorporate their own electrical meters.

The distribution panels are dependent on the TC2 feed different areas of the south (*Princesa Sofía*) and west zones of the building, radiology, physical therapy, data center (CPD), rehabilitation, and air handling units (AHUs). On the one hand, PM5560 meters have been installed in West Zone Module 1, Rehabilitation, CPD, and Radiology 1. On the other hand, PM5110 meters have been installed in Physical Therapy A, South Zone Plants 2–7, South Zone Plants 8–13, Physical Therapy B, Radiology 2, South Zone Elevators and AHUs. Module 3 in TC2 feeds Physical Therapy A, South Zone Plants 2–7, South Zone Plants 8–13, CPD, West Zone Module 1, Physical Therapy B, Radiology 1, Radiology 2, and South Zone Elevators. Module 11 in TC2 supplies electricity to Rehabilitation and AHUs, but it is not measured upstream.

The distribution panels are dependent on the TC3 feed for each of the five Petra air-cooled chillers. On the one hand, Module 3 in TC3 supplies electricity to air-cooled chillers 1–3 and on the other hand, Module 5 in TC3 feeds the remaining air-cooled chillers (4–5). These chillers contain integrated electrical meters that are used.

**Figure 3:**
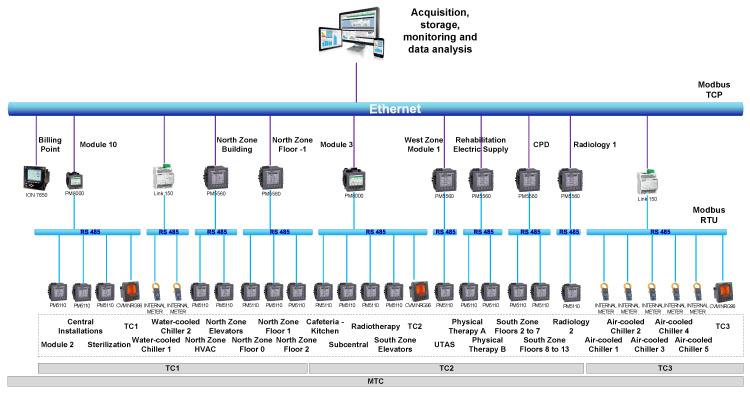
Submetering communication network.

All the electrical meters are linked through a communication network in order to capture and store all data in a database. StruxureWare Power Monitor Expert software by Schneider Electric is used for that task. The submetering network is based on Modbus protocol and combines both Ethernet and RS-485 networks. In order to link Modbus TCP/IP and Modbus RTU networks, a dedicated gateway (Schneider Electric Link 150) and two types of meters with communication capabilities (PM8000 and PM5560) are used. These meters, besides measuring and capturing electrical data, work as gateway and can communicate data directly with the software and the database. On the contrary, PM5110 meters with basic metering functionality require a gateway to communicate with the software and the database. All of these basic meters are connected to the closest gateway in the building. All the databases storing data from the meters are synchronized with the servers managed by the SUPPRESS research group which are located at the University of León. This connection is made through a VPN during the night to reduce the network load. This way, data are made accessible for the calculation server of the research group. Figure 3 summarizes the submetering communication network at the León University Hospital.

**Figure 4:**
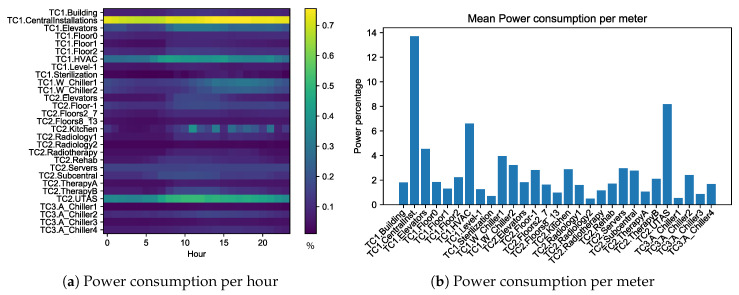
Mean power consumption percentage per hour and per meter, to analyze the contribution of each meter to the whole building consumption.

That submetering network provides a huge volume of data for the analysis which can be biased by high consumption areas. Figure 4 shows two visualizations using the mean values of the power consumption during one year. Figure 4a is a heatmap whose values are the mean percentage of the whole building power consumption for each meter and hour. This figure does not show clearly the hours when meters have stronger influence in the whole consumption because meters with higher consumption overshadow the consumption peaks of the other meters. Figure 4b reveals that there are two meters that use nearly 25% of the energy of the whole building, so these two meters mask the influence of the other meters. For this reason, it is necessary to propose a modeling technique that maximizes the acquisition of relevant knowledge to understand the whole building consumption, being able to effectively model the power consumption peaks instead of just discovering which meters provided a higher consumption.

## 4. Methodology for Feature Extraction

The proposed methodology is based on a deep convolutional autoencoder network which lets us extract relevant information from the submetering network. In this work, the input layer and the output are fed with different data sets that are assumed to provide the same information. Taking advantage of the submetering network, input data are the disaggregated power consumptions, whereas output data contain the total power consumption in the whole building, measured at the main supply (MTC). The purpose is to understand the influence that each hour, meter, or environmental variable has in power consumption through the analysis of the weights of the network. This information can be visualized in plots and graphs so that it can be easily understood.

### 4.1. Dataset

Although the submetering network has different levels, we only use the MTC meter, which records the power consumption of the whole building, and all the endpoint meters installed in different circuits of the Hospital. In order to analyze the influence of each meter, the meters at the lowest submetering layer are used as input to the convolutional autoencoder, whereas the meter of the top layer is the output. The meters located in intermediate layers were not considered because they do not provide additional interesting information and the noisy signals could reduce the performance of the model.

Consumption data are collected and arranged so that the input space contains a daily profile per meter. For that reason, the input space is a three-dimensional tensor or array whose dimensions are D×H×N, where:

- *D* is the number of days for which data were collected.
- *H* is the number of samples per day.
- *N* is the number of meters, from the bottom layer (3) of the submetering network.

In the particular case of the submetering infrastructure under study, the input space would include the measurement from all the meters at the Level 3, with the exception of the *Air-Cooled Chiller 2* that is always off. As a result, in this case, N=29 (see Table 1 for the architecture of the submetering network).

The output layer comprises the consumption daily profiles from only one meter (MTC), so data are arranged in a matrix whose dimensions are D×H.

### 4.2. Approach

Since we are dealing with an unsupervised learning problem, the main idea is to create a network that learns with the information provided by the meters of different levels. For that matter, an autoencoder network is needed. The autoencoders generally aim at replicating in the output layer the same values of the input layer. However, as in this case, there are several applications where the values of the input and the output layers are different, such as noise removal by denoising autoencoders [33], where noise is added to the input so that the network can learn the main information and erase all additional noise. Another one is dimensionality reduction by generalized autoencoder [37], which is used to reconstruct a set of instances rather than itself.

In the proposed approach, the input layer is built as a parallel structure fed with each submeter, whereas the output layer results from the sum of the input data. Therefore, each sample of the output layer follows the equation:

(1) $${P_{D,H}}^{MTC}≃\sum_{i=1}^{N}{P_{D,H}}^{\mathrm{Me}i},$$

where ${P_{D,H}}^{\mathrm{Me}i}$ denotes the power consumption of one submeter (it corresponds to a meter on the level 3 of the submetering architecture), for a sample time *H* of a certain day *D*. The sum of all *N* submeters installed in the low level (3) of the submetering architecture is approximately the same as the measurement of the top level meter MTC, where power is denoted as ${P_{D,H}}^{MTC}$. That sum is not exactly the same, due to the losses and noise in the submetering network [38]. The autoencoder is not only expected to provide relevant information through the analysis of the bottleneck neurons, but it should also remove noise from the measurements in the grid.

In order to achieve feature extraction, the autoencoder includes convolutional layers since they have already been used efficiently to obtain shared weights and find effective features [27]. Since one dimension of input data is temporal (*H*), 1D convolutions are used and each meter is treated as a different channel, so the proposed method has to deal with parallel inputs. This setup is analogous to that used by some image processing methods, which process separately the convolutions for three different colors and then merge the results. However, our method uses separable convolutions in order to obtain the weights of the different input channels instead of averaging them.

**Figure 5:**
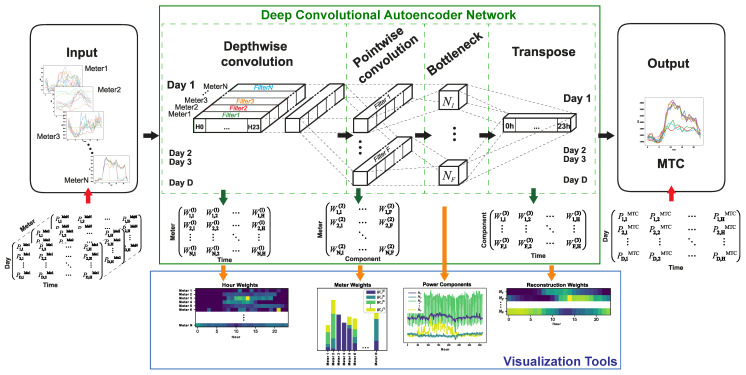
Feature extraction using a convolutional autoencoder.

Figure 5 shows the proposed methodology that uses a four-layer network. It depicts the structure of the deep convolutional autoencoder, the input and output data structure, and different features about the submetering network that are learnt in each layer. It makes use of an autoencoder where the encoding part is built as a separable convolution [29], which comprises two steps: the first convolution learns the weights of the signal for each channel and the second one learns the weight of the channel itself. Let us describe each layer and its purpose in detail:

- *Depthwise convolution* is the first layer, where a 1D convolution (N×H) is applied to the daily profile of each meter. Thus, this layer obtains a two-dimensional matrix of weights which will provide information about the influence of each sample time of the meter in the whole building consumption. That matrix is:
  (2) $$\left(\begin{array}{cccc} W_{1,1}^{\left(1\right)} & W_{1,2}^{\left(1\right)} & \cdots & W_{1,H}^{\left(1\right)}\\ W_{2,1}^{\left(1\right)} & W_{2,2}^{\left(1\right)} & \cdots & W_{2,H}^{\left(1\right)}\\ \vdots & \vdots & \ddots & \vdots \\ W_{N,1}^{\left(1\right)} & W_{N,2}^{\left(1\right)} & \cdots & W_{N,H}^{\left(1\right)} \end{array}\right)$$
  and it can be visualized in a colormap where each pixel provides information about the time feÓature. The upper number of the weights denotes the layer number. Although the number of filters of this layer could be higher than one, it is only one per meter since the proposed method tries to obtain interpretable results. Therefore, the total number of filters that are learnt is *N*. Furthermore, the dimension of the filter is the same as the number of time samples in order to obtain one weight related with each time sample.
- *Pointwise convolution*: the second layer is another convolution carried out in the meters dimension, so the weights of this convolution represent the influence of each meter in the building consumption. The number of filters that are used in this pointwise convolution sets the dimension of the latent space of the autoencoder. The following weight matrix is obtained:
  (3) $$\left(\begin{array}{ccc} W_{1,1}^{\left(2\right)} & \cdots & W_{1,F}^{\left(2\right)}\\ W_{2,1}^{\left(2\right)} & \cdots & W_{2,F}^{\left(2\right)}\\ \vdots & \ddots & \vdots \\ W_{N,1}^{\left(2\right)} & \cdots & W_{N,F}^{\left(2\right)} \end{array}\right)$$
  where the dimensions of the matrix are the number of meters *N* and the number of neurons of the bottleneck *F*. These weights can be plotted in a bar char, showing the important features of each meter.
- *Bottleneck*: this layer is the output of the previous one and defines the low-dimensional latent space where relevant information about the power consumption of the building is obtained. Thus, if these data are correlated with environmental variables, this information is related to their influence in each meter through the weights of the previous layer. Since the dimension of this layer is configurable, it becomes one of the most important parameters of the method, i.e., the number of components of the power consumption. When the autoencoder is trained, the output of this bottleneck layer gives the main daily components of the power consumption.
- *Transpose convolution*: Finally, the last layer is the decoder phase of the autoencoder. There is only one output channel, so only one convolution is needed to obtain the consumption profile of the main supply meter. Although the appropriate operation at this layer would be the inverse convolution, there exists no such operation available. Therefore, a transpose convolution is used as a mirror operation in a convolutional autoencoder [39]. This transpose convolution also provides a weight matrix:
  (4) $$\left(\begin{array}{cccc} W_{1,1}^{\left(3\right)} & W_{1,2}^{\left(3\right)} & \cdots & W_{1,H}^{\left(3\right)}\\ \vdots & \vdots & \ddots & \vdots \\ W_{F,1}^{\left(3\right)} & W_{F,2}^{\left(3\right)} & \cdots & W_{F,H}^{\left(3\right)} \end{array}\right)$$
  where each component provides information about the temporal influence of each component of the latent space. The dimension of the matrix is the number of neurons of the latent space *F* and the number of time samples in the daily profile *H*.

In short, each layer of the trained network can be used to explore different aspects of the building behavior. The *Depthwise convolution* provides information about the consumption of each meter with respect to the hour. The *Pointwise convolution* is a decomposition of the whole power consumption into a number of components equal to the number of filters. The study of the correlations between these components and the environmental variables might let us better understand the factors behind consumption. Interesting environmental variables are the ambient temperature, with an obvious influence on the HVAC consumption, and the day of the week, which is linked to human activity, i.e., occupation and working patterns. In addition, finally, the weights of the *Transpose convolution* are related with the daily hours in which these factors have more influence in the whole building consumption.

To train the model, a constraint on non-negativity is used in the layers. Since the main purpose of the network is the analysis of the neuron weights in each layer and we are dealing with power consumption, a negative coefficient could not be explained because it would be interpreted as negative power consumption, i.e., as a power generating node. The Hospital that is studied does not have such installations so a negative component is forbidden. It needs to be noted that, if this restriction is not used, the training error of the network is reduced, but the interpretation of the results would certainly lead to wrong conclusions. Another constraint used in training is that bias will be equal to zero in all layers. It is based on the assumption that any non-registered consumption should remain proportional to the measured one. This assumption is confirmed by the training of the network since the training error increases when a non-zero bias is calculated.

The criterion used to select the parameters of the deep neural network is the minimization of the reconstruction error. This error is computed as the difference between the real measure of the MTC and the signal that is generated by the network when the input data are fed. It is evaluated using the MAPE (Mean Absolute Percentage Error) since it is widely used to measure performance of algorithms that estimate power consumption [40]:

(5) $$MAPE=\frac{100}{n}\sum_{i=1}^{n}\left|\frac{{P_{D,H}}^{MTC}-{\hat{P}{}_{D,H}}^{MTC}}{{P_{D,H}}^{MTC}}\right|$$

where ${P_{D,H}}^{MTC}$ denote the actual power consumption of the whole building at the top level meter MTC for the time *H* day *D* and ${\hat{P}{}_{D,H}}^{MTC}$ is the power estimated by the deep autoencoder of the MTC meter for the same time.

## 5. Experimental Results and Discussion

### 5.1. Experiment

In order to assess its usefulness for feature extraction, the proposed approach is applied to a dataset acquired from the submetering infrastructure of the León University Hospital, which was described in Section 3. The results to be analyzed would be the weights obtained for the depthwise, pointwise, and transpose convolution that, as explained before, are expected to provide information about different aspects of consumption.

The dataset was collected from the submetering structure during one year, from March of 2018 to February of 2019, with a sampling rate of one minute. Data were resampled to one hour in order to reduce metering noise and occasional peaks. As explained in the previous section, only the meters corresponding to the lowest submetering layer and the MTC meter are considered. As a result, the input dataset has 365 samples of 24 hours and 29 power meters (from level 3 of the architecture), whereas the output dataset has 365 samples of 24 hours for a single meter (the MTC meter of the Level 0).

The deep neural network was implemented in *Python* using *Keras* [41]. This library is a high-level API running on top of *TensorFlow* [42], which is an open source platform to create and train neural networks. Both libraries make it easy to train and test different architectures of convolutional autoencoders. In addition, they can run on a *GPU* (Graphics Processing Unit), exploiting parallel computing so that models are calculated faster, allowing the use of these techniques in almost real time.

**Figure 6:**
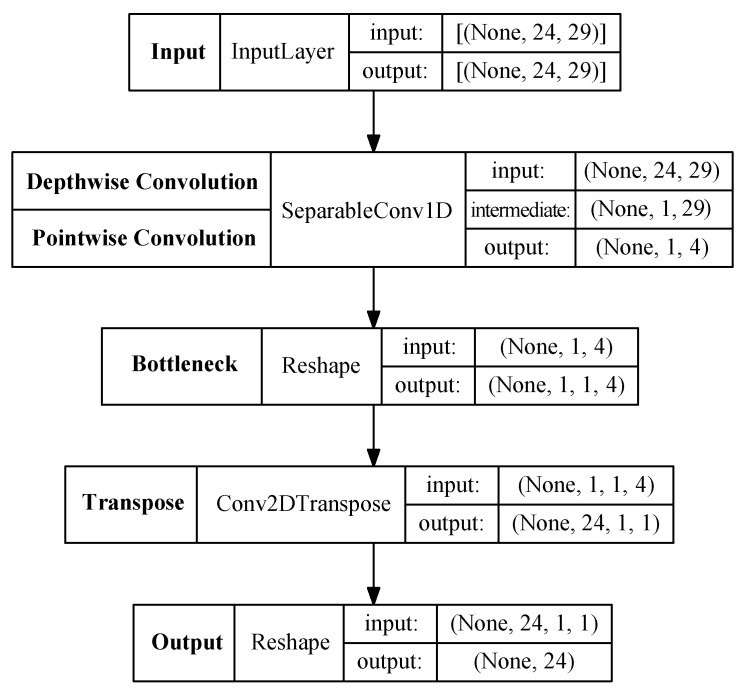
Structure of the convolutional autoencoder used to extract the features from the meters.

Figure 6 summarizes the dimensions and types of layers that were used to implement the proposed architecture. It must be noted that function names in *Tensorflow* differ from the notation used in the document, which has been kept in bold, and that some adjustments need to be made. Since there is no transpose function in *Tensorflow* for the 1D convolution (needed in the encoder phase), a 2D convolution is used as transpose, but using a kernel size of (24,1) so that it is equivalent. It is necessary to add a dimension to the data structure so that the output of a 1D Convolution is suitable for a 2D transpose. For that reason, a *Reshape* layer is added to the model in the bottleneck. Finally, another reshape layer is added to feed the output so that the model structure matches the data structure of the MTC power data. The purpose of this layer is only to remove the additional dimensions that are created by the Conv2D Transpose function.

**Figure 7:**
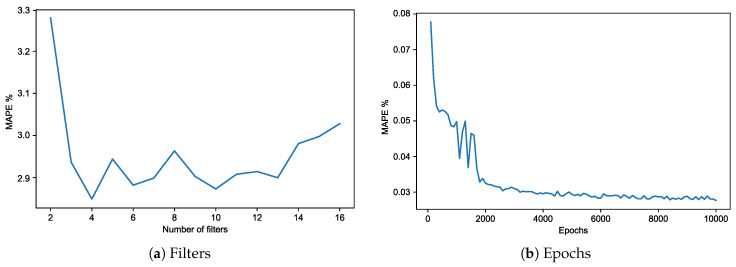
Training error (MAPE) of the model.

All the data used in the training phase are used to compute the performance metric. Note that the proposed approach does not aim at providing the most accurate regression. Instead, feature extraction is the ultimate target of this approach, i.e., the approach solves an unsupervised problem. The most important parameter that must be tuned is the number of convolution filters because it sets the number of neurons in the bottleneck layer. As stated in the previous section, the MAPE is used to tune the model (See Figure 7). The lowest value (2.81 %) is reached when the number of filters has been set to 4 (See Figure 7a) and the number of epochs to 5000, since the error remains almost constant from this point on (See Figure 7b).

### 5.2. Results from the Depthwise Convolution

**Figure 8:**
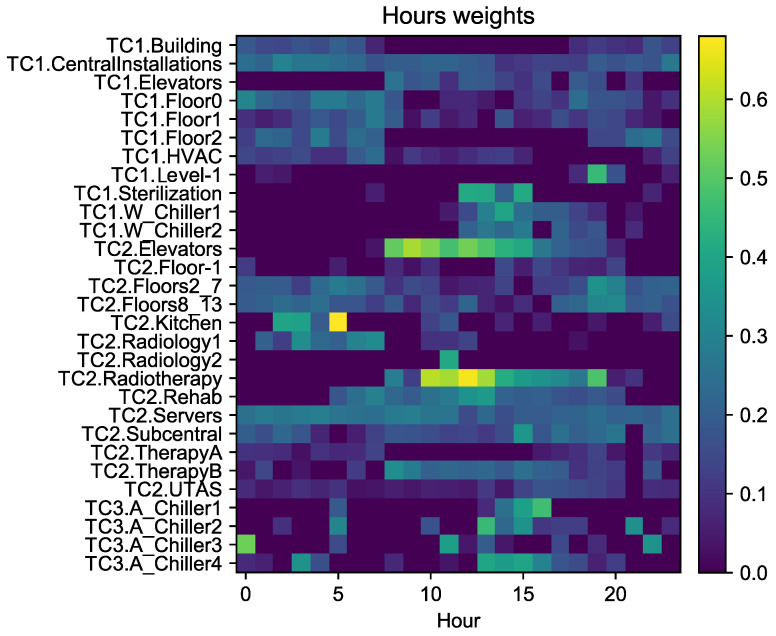
Heatmap showing the weights of the depthwise convolution. Blue colors correspond to low values near zero and yellow ones to high values.

The hourly influence of the meters can be obtained by analyzing the weight of the *Depthwise convolution*. Since we are using a filter of dimension 24 and the output is only one neuron per meter, there is a weight matrix of dimension 24 × 29 that can be visualized as a heatmap in which the color is the value of the weight of the convolution. Figure 8 shows different behaviors depending on the meter:

- Meters whose weights are high during the central hours of the day such as *TC2.Elevators*, *TC2.Radiotherapy*, or *TC1.Elevators*. Those meters measure power consumption of building zones that are used more intensively during the day. The increase of the consumption of these areas has a strong impact on the consumption of the whole building. Since some of them include large equipment such as medical machines that create high consumption peaks, they change the MTC power profile.
- Meters whose weights are approximately constant throughout the day such as *TC2.Servers* or *TC1.CentralInstallations*. Those meters measure support machines and infrastructure that work all day. At any time, there might be fluctuations in the consumption due to the start and stop of different equipment.
- Meters that record power consumption of chiller machines such as *TC1.W_Chiller* or *TC3.A_Chiller*. The weights are higher during the afternoon, coinciding with the warmest hour of the day because chillers usually start and increase the consumption at those hours. The *TC3.Chillers* show some high weights without a well defined pattern during the night. Those upturns are due to machine rotation that takes place during the night when they are powered off due to low ambient temperature and later others are started to balance the operation time of the machines. Since chiller starts demand a lot of power, they have a strong influence on the global consumption and so the network gives them high weights.
- Particular meters such as *TC2.Kitchen* or *TC1.Level-1* that have more influence in a specific hour at night. This behavior is due to the quite low consumption of these zones. In the central hours of the day, the relative importance of this consumption is less significant than during the night when the consumption of the whole building is lower.

### 5.3. Results from the Pointwise Convolution

**Figure 9:**
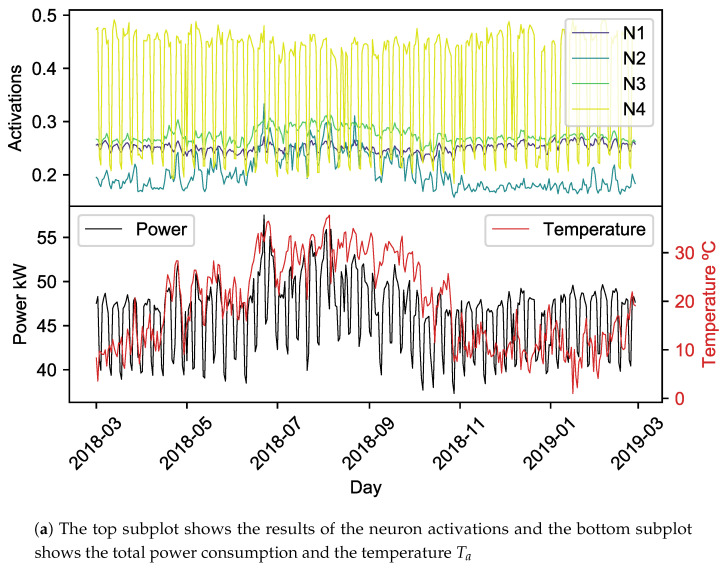

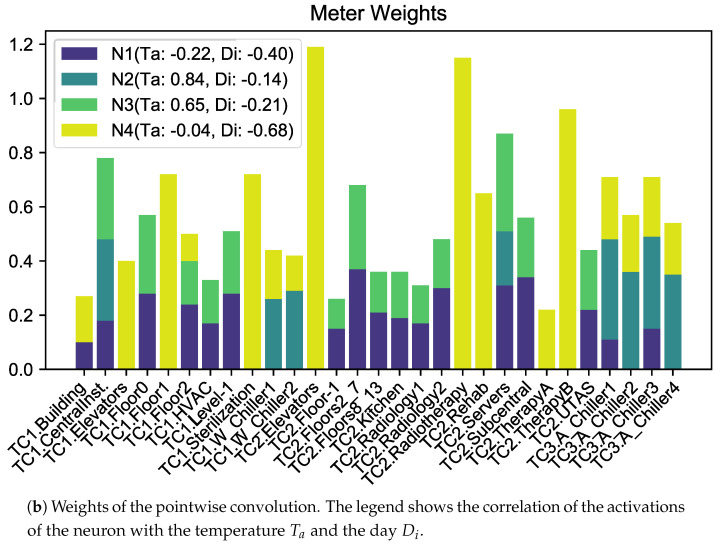
Results of the pointwise convolution (weights and activations).

After the depthwise convolution, the input of the pointwise phase is comparable to a model of daily consumption of the meter, so the result of the activation of the pointwise neuron can be analyzed as a daily consumption. The result of pointwise convolution reduces the input dimension (29) to the number of neurons set in the bottleneck, 4 in this case (See Figure 9). These four neurons (N1, N2, N3, N4) need to maximize the storage of information about the building consumption. The activation signals that result from the decomposition of the daily power consumption can be understood as power components. In order to interpret the factors behind consumption, it is interesting to study the correlation between the daily components of the power activation and the daily mean values of environmental variables. The variables that have been chosen are the ambient temperature $T_{a}$, obtained from a temperature sensor, and the weekday $D_{i}$, converted to a variable whose values are between 1 for Mondays to 7 for Sundays. Note that workdays are related to low values and the weekend to high ones. The top plot of Figure 9a shows the values of the activations for each day enabling the comparison with the value of the temperature and the whole building consumption that is shown in the bottom plot of this figure. Figure 9b shows the weight of each neuron with a bar chart, encoded in different colors. Furthermore, the correlation between the component created by the neuron and the environment variable is shown in the legend of the plot. Values of correlation near 0 show that the corresponding power component is not influenced by this environmental variable, whereas values near to −1 or 1 denote high dependency. The results that can be obtained are:

- **Neuron 1 (N1)**: this power component is inversely correlated to temperature and directly correlated to weekday. It can be seen in the bar chart that the meters related to this neuron are mostly the ones measuring the installation related to the hospital rooms. This power component would then model the consumption of the support systems that provide heat to the building.
- **Neuron 2 (N2)**: it codifies a power component that is only highly correlated with the temperature since its correlation with this variable is 0.84. In the activation plot (orange line), it can be seen that the value is higher in summer days than in winter ones. The bar plot of the weights leads us to think that the meters that compose this power component are all the chillers, the central installations and the servers. This result is rational, since this neuron is related to the circuits that have equipment related to the cooling systems.
- **Neuron 3 (N3)**: it codifies a power component with a high correlation with temperature and low correlation with the weekday. It is related to the hospital rooms and its support equipment, but it also models in part the consumption related to the cooling systems since its consumption is higher during the summer.
- **Neuron 4 (N4)**: it is a neuron highly correlated with the weekday (–0.68). The plot line shows that this power component has a periodic behavior of seven days that can be related to the workday, when the value is high, and the weekend, when the value is low. This neuron is the only weight of meters such as North Zone and South Zone Elevators, Radiotherapy, etc. This finding indicates that these circuits, as the tag points out, measure the consumption of equipment whose use highly depends on the building occupation. The activity in those areas during the weekends is low. Other meters such as the chillers are weighted by this neuron, showing that these meters also have higher consumption on the days that the activity of the building is high.

As it can be seen in the bar chart (Figure 9b), the pointwise convolution discovers basically three types of circuits. The ones that only have a neuron 4 component are the circuits related to medical equipment and systems that strongly depend on the building occupation. The circuits related to the cooling systems whose consumption is higher during the summer. In addition, the meters that have weights in the neuron 1 and 3 and that are related to the rooms and the support systems, so they have fewer consumption peaks and the consumption is more and less constant.

### 5.4. Results from the Transpose Convolution

**Figure 10:**
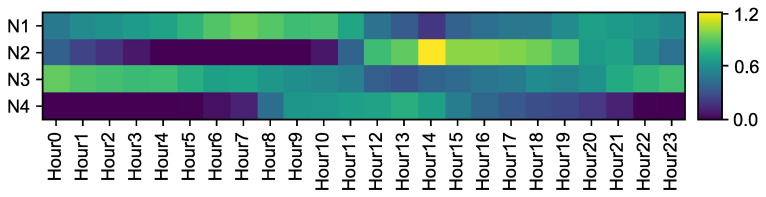
Heatmap showing the weights of the transpose convolution.

The last convolution produces 24 components so that the daily profile can be reconstructed again. Using the same criteria as in the depthwise convolution, a heatmap with the weights of the transpose convolution can be created with the weights of the layer. The weights are shown in Figure 10. This visualization complements the information provided by the pointwise convolution, since it gives information about the influence during each hour of the components calculated. Blue colors correspond to lower weights and yellow ones to higher values. It can be seen in the figure that:

- The first neuron, which is weakly correlated with temperature and weekday, shows a similar color during all the day hours. This validates the previously deduced idea that this neuron stores information about the base consumption of the circuits of the building.
- The second neuron is the one that reflects the consumption related with cooling systems, since it is highly correlated with temperature. As it can be seen in Figure 10, weights are higher during the afternoon, which is consistent with the use of cooling machines which are usually powered on during the afternoon.
- The third neuron, similarly to the first one, shows the same influence throughout the day. Its correlation is higher with respect to the temperature so it stores information about base consumption during the summer months.
- The last neuron shows high weights during the central hours of the day and low values during the night. This influence is a consequence of the high correlation of this neuron with the weekday, so it keeps the information related to the building activity.

### 5.5. Discussion

The knowledge acquired by means of a simple analysis is scarce, since the meters with high consumption hide the features of those with low consumption. However, the proposed methodology provides detailed information about the main peak hours of each meter and its relations with other meters by analyzing the results from the different steps of the methodology.

The weights of the depthwise convolution (H×N) provide information about the hourly influence of each meter. It is possible to distinguish meters measuring consumptions that remain approximately constant from those with a strong dependence on the time of the day. Among the time-dependent meters, it is possible to find meters with higher consumption during the day, in the afternoon or at night. For example, the depthwise convolution shows that the elevators work more often during the day, whereas there are other areas whose consumption is fairly distributed throughout the whole day, such as support equipment (e.g., servers). The understanding of these behaviors could be useful for a more efficient planning of activities in the building.

The weights of the pointwise convolution can be understood as power consumption components or factors and can be therefore correlated with environmental variables. Since the behavior of each meter is explained by means of a different combination of these components, the correlation values show the influence of the environment in each area of the building. Among the four components that are obtained, two are strongly correlated with only one environmental variable (temperature and day of the week, respectively) and two express a mixed influence of both environmental variables. When those factors are related to particular meters, it can be seen that the results can be intuitively explained from prior knowledge, e.g., associating the temperature-dependent component with the meters that measure chillers consumption or associating the weekday-dependent component with the meters of elevators or areas subject to schedules of the medical consultations. In addition, the correlation shows meters that have high dependence with temperature, such as the chillers, and others that are related with the baseline consumption, such as the central installations, allowing us to know which meters have a constant consumption and which ones show a periodic behavior. Despite the usefulness of these findings being limited for experienced staff, we argue that they could be valuable to assess the quality of the decomposition.

Finally, the weights of the transpose convolution let us study the relation between the components obtained by the pointwise convolution and the hours, providing information about the hourly behavior of the fundamental components which are obtained in the previous step of the methodology. It can be seen that weights are higher during some hours of the day only for some components. If these results are analyzed along with those from the pointwise convolution, it is also noticeable that those components whose consumption varies the most throughout the day are the ones related with chillers consumption and areas with specific operating hours. These findings could be useful for the consumption peak flattening.

## 6. Conclusions

A new methodology is presented in this paper to take advantage of a submetering architecture for a better understanding of the energy use patterns in large buildings. This methodology is able to find complex relations between the meters, the whole building consumption, and environmental variables. These relations provide information about the influence of the day hour in the peak consumption of each meter. All of this information is easily analyzed by means of simple visualizations which are provided by the proposed methodology. The model is based on a convolutional autoencoder architecture that reconstructs the whole building consumption with information provided by the submetering network. It makes use of a separable convolution that is composed of a depthwise convolution, which extracts the information about the influence of the hour in the consumption of each meter, and a pointwise convolution, which learns the influence of each meter. This information is extracted from a low dimensional space which is obtained by the autoencoder. Finally, the model reconstructs the consumption by means of a transpose convolution.

The methodology is tested with a real submetering architecture deployed at the León University Hospital. The data used is active power consumption of 30 meters (29 submeters and 1 global meter) during one year, so that analysis covers different seasonal dynamics. Furthermore, other sensor measurements such as the ambient temperature are considered in order to obtain the influence of external variables on the consumptions. The model is tuned by means of the reconstruction error of the convolutional autoencoder. The main parameter to be adjusted is the number of neurons of the middle layer, which was set to 4. Once the model is trained, the visualizations show different consumption patterns of the building areas.

The proposed methodology has demonstrated that it is possible to extract complex information from the power meters easily, avoiding the problems caused by the different scale of the consumption of each meter, and visualize it, using the data from the same model without using other methods, making the methodology simpler. To sum up, this methodology provides, with the same neural network, a power decomposition, an environmental influence analysis, a peak hour analysis, and clustering information, all with its correspondent visualization.

## Figures and Tables

**Table 1:** List of the electrical meters and submetering architecture at the León University Hospital.

Level 0	Level 1	Level 2	Level 3
MTC	TC1	Module10	North Zone Floor −1
			North Zone Floor 0
			North Zone Floor 1
			North Zone Floor 2
			North Zone Elevators
			North Zone Building
		Module2	Water Cooled Chiller1
			Water Cooled Chiller2
			North Zone HVAC
			Sterilization
			Central Installations
	TC2	Module 3	Physical Therapy A
			Physical Therapy B
			South Zone Floors 2–7
			South Zone Floors 8–13
			South Zone Elevators
			CPD
			West Zone Module 1
			Radiology 1
			Radiology 2
			Radiotherapy
			Cafeteria-Kitchen
			Subcentral
			Rehabilitation
			AHUs
	TC3		Air Cooled Chiller 1
			Air Cooled Chiller 2
			Air Cooled Chiller 3
			Air Cooled Chiller 4
			Air Cooled Chiller 5

## Abbreviations

The following abbreviations are used in this manuscript:

NILM	Non-Intrusive Load Monitoring
CNN	Convolutional Neural Network
AE	Autoencoder
DAE	Denoising Autoencoder
SAE	Stacked Autoencoder
IoT	Internet of Things
HVAC	Heating, Ventilating, and Air Conditioning
BMS	Building Management System
MTC	Main Transformation Center
